# Tuning substrate temperature for enhanced vacuum-deposited wide-bandgap perovskite solar cells: insights from morphology, charge transport, and drift-diffusion simulations†

**DOI:** 10.1039/d5el00021a

**Published:** 2025-05-14

**Authors:** Lidón Gil-Escrig, Jasmeen Nespoli, Fransien D. Elhorst, Federico Ventosinos, Cristina Roldán-Carmona, L. Jan Anton Koster, Tom J. Savenije, Michele Sessolo, Henk J. Bolink

**Affiliations:** a Instituto de Ciencia Molecular, Universidad de Valencia C/Catedrático J. Beltrán 2 46980 Paterna Spain lidon.gil@uv.es michele.sessolo@uv.es; b Instituto de Física del Litoral (IFIS-Litoral), CONICET-UNL Güemes 3450 S3000GLN Santa Fe Argentina; c Department of Chemical Engineering, Faculty of Applied Sciences, Delft University of Technology 2629 HZ Delft The Netherlands; d Zernike Institute for Advanced Materials, University of Groningen Nijenborgh 3 9747 AG Groningen The Netherlands

## Abstract

The efficiency of vacuum-processed perovskite solar cells lags behind that of solution-processed devices, partially because of the limited spectrum of deposition parameters that can be controlled during deposition. Substrate temperature is in principle a powerful tool to control the condensation and crystallization of thin films, but has been scarcely investigated for perovskites. This study systematically investigates the effect of substrate temperature on the deposition of the wide-bandgap perovskite Cs_0.2_FA_0.8_Pb(I_0.8_Br_0.2_)_3_. We observe temperature-dependent morphological changes linked to variations in the adhesion coefficient of formamidinium iodide. Optical, structural, and optoelectronic analyses reveal that increasing the substrate temperature from −20 °C to 75 °C enhances charge carrier mobility and recombination lifetime by an order of magnitude. However, these improvements do not directly translate into better device performance due to competing factors such as morphology, interface energetics, and trap densities. Using drift-diffusion simulations, we identify key performance-limiting parameters, including ion mobility and charge trapping at interfaces and in the bulk. By optimizing the organic/inorganic deposition rate at −20 °C, we achieve state-of-the-art efficient wide-bandgap perovskite solar cells with enhanced thermal stability. This study highlights substrate temperature as a crucial parameter for improving material quality and device performance in vapor-deposited perovskites.

## Abstract 2

Substrate temperature governs precursors adhesion, film morphology, and charge transport in vacuum-deposited perovskite films. Optimized growth at −20 °C yields efficient, thermally stable wide-bandgap perovskite solar cells.

## Graphical abstract

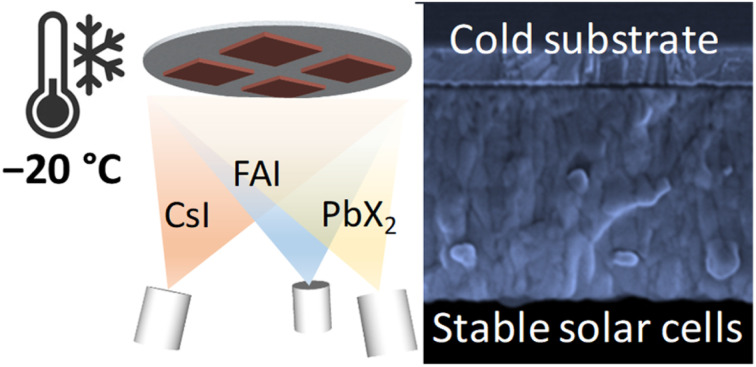

**Broader context**

The rise of perovskite solar cells has the potential to reshape the landscape of next-generation photovoltaics, offering high efficiency and cost-effective fabrication. While solution processing dominates perovskite research, industrial-scale semiconductor manufacturing largely relies on vacuum-based deposition methods. Unlocking the full potential of vapor-deposited perovskites is therefore important for bridging the gap between laboratory advances and commercial deployment. In this work, we systematically explore how substrate temperature influences the growth, composition, and optoelectronic properties of wide-bandgap perovskite films, a key step toward optimizing vapor-phase fabrication. Our findings reveal a significant enhancement in charge carrier mobility and recombination lifetime, along with insights from drift-diffusion modeling that identify performance-limiting factors. By optimizing deposition conditions, we demonstrate efficient and thermally stable perovskite solar cells, highlighting the untapped potential of substrate temperature control in vapor-processed perovskites.

## Introduction

Perovskite solar cells have rapidly emerged as a promising technology for next generation photovoltaics (PVs).^1–3^ This is a consequence of the properties of metal halide perovskite semiconductors (herein simply perovskites), such as the sharp absorption edge and high absorption coefficient (consequence of their direct bandgap, *E*_g_), long charge diffusion length and lifetime, and defect tolerance.^4–10^ An important characteristic of perovskites is the ability to finetune their bandgap *via* compositional substitution.^11–14^ This property is not unique to perovskites: the bandgap of III–V semiconductors, for example, can also be varied by several electronvolts by alloying within the group III or V of the periodic table.^15^ In perovskites, however, the chemical synthesis and substitution can be carried out *via* low energy synthetic processes.^16^

Perovskite thin films can be deposited *via* solution processing or by vapour-based techniques. The vast majority of the literature available on the topic relies on solution-processed materials, while vapour-deposited perovskite films and devices have been investigated only by a small number of research groups. On an industrial scale, however, most semiconductors are prepared using dry, vacuum-based methods. This is the case for CdTe thin-film solar cells, representing roughly 5% of the world PV market, fabricated by vapour-transport deposition.^17^ Hence the development of vacuum- and vapour-based techniques in the processing of perovskite films and solar cells can be beneficial for an easier transition from the lab to the industry.^18–20^ Additionally, there are other benefits of vapour-based deposition techniques applied to perovskites, such as the high level of control over the film thickness for uniform large-area deposition,^21–24^ purity (solvent-free) of the material, low temperature processing, conformal coating, and the straightforward fabrication of multilayer devices. However, the power conversion efficiency (PCE) of vacuum processed perovskite solar cells lags behind that of solution-processed devices,^25^ which have demonstrated PCE exceeding 26%.^26^

As of now, the most efficient devices with vacuum co-evaporated perovskites have PCE only slightly above 20%,^27–30^ with only a few reports on sequential vacuum deposited perovskites with higher PCE.^31,32^ This gap is related with the limited spectrum of deposition parameters that can be controlled during vapour deposition, namely base pressure,^33,34^ type and number of chemical precursor,^35–37^ deposition rate,^38,39^ substrate type^29,40^ and substrate temperature.^29,41,42^ The latter is in principle a powerful tool to control the condensation and crystallization of thin films, but has been scarcely investigated for perovskites. In the pioneer work of Liu *et al.*,^43^ the perovskite was deposited with a controlled stage temperature of 21 °C. In subsequent works, the substrate was kept at higher temperature than ambient (50 °C),^44^ but no specific justification for the choice of the temperature was provided.

The first study to systematically screen the effect of the substrate temperature on the co-evaporation of methylammonium lead iodide (MAPI) was reported by Wang *et al.*, where the temperature was varied from −50 °C to 110 °C.^45^ At low temperature (−50 °C), the film was found to be non-uniform and rough. At 20 °C, the films showed a full coverage with uniform and flat morphology, which was however expected as the deposition process was previously optimized at this same temperature. Larger grains but less uniform morphology was observed at higher substrate temperatures (80 °C and 110 °C). The changes in the film morphology and degree of conversion were ascribed to the temperature-dependent sticking coefficients *s* of MAI on the substrate.

The general expression for the sticking coefficient contains a temperature-dependent Boltzmann term, exp(−*E*_act_/*k*_B_*T*), where *E*_act_ is the activation energy for the adsorption process, *k*_B_ the Boltzmann constant and *T* the temperature.^46^ At higher temperature, the adhesion and reaction of MAI on the forming MAPI film would hence be reduced.^47,48^ This observation was further corroborated by Roß *et al.*, who observed that the tooling factor (hence the adsorption rate) of MAI is significantly influenced by the temperature of the substrate, while only minor changes were seen for PbI_2_.^29^ Lohmann *et al.* also investigated the formation of MAPI films by co-evaporation of its precursors for substrate temperatures between −2 °C and 23 °C.^41^ In spite of the limited temperature range, they observed a clear dependence of the film morphology on substrate temperature, with large, micrometre-sized grains formed at low temperature (−2 °C) and smaller grains appearing at 23 °C. In these examples, the temperature-dependent MAI adhesion lead inevitably to alteration of the perovskite stoichiometry at high and low temperature, with a temperature window for optimal perovskite deposition close to room temperature (RT).

In this manuscript, we systematically studied the effect of the substrate temperature on the deposition of a more complex material, the wide bandgap perovskite Cs_0.2_FA_0.8_Pb(I_0.8_Br_0.2_)_3_, where FA is formamidinium. Similar to previous reports, the morphology of the film is found to be considerably temperature-dependent, which is here ascribed to the temperature-dependent adhesion coefficient of the organic precursor formamidinium iodide (FAI). We observe clear differences in the material properties, that are investigated by optical, structural and optoelectronic techniques. In particular, the effective mobility of the mobile charge carriers and their recombination lifetime were found to increase by one order of magnitude when increasing the substrate temperature from −20 °C to 75 °C, according to changes in composition and structure of the films. These properties are not directly translated to an improved device functioning, as other parameters (morphology, substrate-dependent growth, interface energetics) contribute to the performance of the perovskite solar cells. Consequently, we employ drift-diffusion simulations to identify the parameters that limit their performance. Specifically, the *J*–*V* characteristics at multiple light intensities and substrate temperatures are measured and simulated. We find that not only the charge carrier mobility affects the *J*–*V* characteristics, as the trap densities at both interfaces and in the bulk, the ion concentration and ion mobility, all have an effect on the performance. Finally, using a substrate temperature of −20 °C we optimized the organic/inorganic deposition rate ratio, and obtained efficient wide bandgap perovskite solar cells with enhanced thermal stability. This work shows the potential of the substrate temperature during vapor processing of perovskites to obtain materials and devices with improved quality.

## Results and discussion

We selected the archetypical wide bandgap perovskite Cs_*n*_FA_1−*n*_Pb(I_1−*x*_Br_*x*_)_3_ as it can be applied in tandem solar cells, both in combination with silicon or with another complementary narrow bandgap perovskite.^49–53^ The approximate stoichiometry used as a starting material is Cs_0.2_FA_0.8_Pb(I_0.8_Br_0.2_)_3_, obtained by simultaneous co-sublimation of CsI (0.1 Å s^−1^), FAI (0.45 Å s^−1^) and a mixture of PbI_2_ and PbBr_2_ (0.35 Å s^−1^) at a 1 to 8 molar ratio, a protocol adapted from previous reports.^54,55^ The final thickness of the perovskite film was controlled *via* the quartz crystal microbalance (QCM) sensor controlling the lead halide deposition rate, and the process was interrupted at a reading of 280 nm, corresponding to 500–550 nm thick perovskite film. The substrate temperature was controlled by an oil-cooled copper substrate holder using an external temperature bath, and the perovskite deposition was carried out with substrate temperatures ranging from −20 °C to 75 °C.

**Fig. 1:**
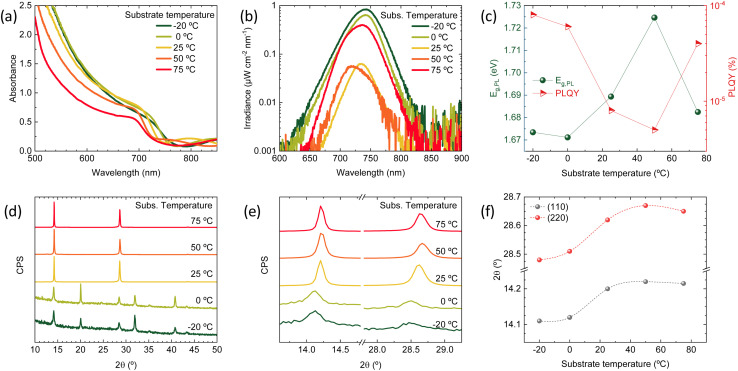
(a) Optical absorbance spectra of a series of Cs_0.2_FA_0.8_Pb(I_0.8_Br_0.2_)_3_ perovskite films deposited with increasing substrate temperature. (b) Calibrated photoluminescence spectra of the same samples recorded upon excitation with a green laser (515 nm) with an irradiance of approximately 50 mW cm^−2^. (c) Bandgap energy extracted from a bi-Gaussian fit of the PL spectrum (left, dark green) and corresponding PLQY (right, red) for the same sample series. (d) XRD patterns for the perovskite films deposited at the different substrate temperatures and (e) zoom of the same patterns on the (110) and (220) diffraction peaks. (f) Trend of the (110) and (220) peak position with increasing substrate temperature (lines are guide to the eye).

Films were prepared on glass substrates and had a dark brown appearance after deposition, without any additional thermal treatment. The optical absorption of films deposited at different substrate temperatures is reported in Fig. 1a. With increasing temperature, a blue shift of the absorption edge from approximately 750 nm to 700 nm can be observed. In parallel, one can observe a diminished absorbance at lower photon wavelength (500–700 nm), which can be due to a lower absorption coefficient or to a reduced thickness. The presence of intense but different interference fringes below bandgap indicates that the thickness is changing (diminishing) with increasing substrate temperature. Both the blue-shift and the reduced thickness for higher substrate temperature suggest a reduced intake of FAI.

The photoluminescence (PL) spectra (Fig. 1b) are also varying in intensity (related to the PL quantum efficiency, PLQY) and spectral position (due to bandgap, *E*_g_, variation) with increasing substrate temperature. However, the trend (Fig. 1c) is not monotonic: at a substrate temperature of −20 °C, the *E*_g_ is approximately 1.67 eV and increases up to and above 1.72 eV when the temperature is raised to 50 °C. At the highest substrate temperature in our series (75 °C), the *E*_g_ drops to approximately 1.68 eV. A similar trend is observed for the PLQY, which decreases from approximately 10^−4^ for the perovskite deposited at −20 °C to 5 × 10^−6^ for the perovskite deposited at 50 °C, and then rises again to 3 × 10^−5^ when the substrate temperature is 75 °C. The PL measurements confirm the trend observed in the optical absorption, with a deviation for the sample deposited at 75 °C. As both the bandgap and the PLQY are simultaneously changing, a better comparison between the series of materials can be done calculating the ratio of the quasi-Fermi level splitting (QFLS) estimated from the PLQY to the QFLS in the radiative limit (PLQY = 1) at each bandgap. As shown in Fig. S1,† the ratio diminishes from 0.87 to 0.82 when the substrate temperature is varied from −20 °C to 50 °C, and increases to 0.86 for the perovskite deposited at 75 °C. This implies an increase of the non-radiative recombination rates from −20 °C to 50 °C, and a partial recovery at 75 °C.

The series of perovskite films was also analyzed by X-ray diffraction (XRD, Fig. 1d). At low substrate temperature (−20 °C and 0 °C) similar XRD patterns were obtained, with all diffraction peaks in accordance with a randomly oriented tetragonal perovskite structure (Fig. S2†). By increasing the substrate temperature (≥25 °C), the diffractograms change substantially. First, the signal-to-noise ratio (SNR) was found to be three orders of magnitudes higher in the high substrate temperature deposited films, (raw data in Fig. S3†). The less intense and broader peaks observed in the films that were deposited at low temperature suggest a higher degree of disorder, due to the smaller grains (larger grain boundaries surface) and/or amorphous phases. This might indicate the presence of amorphous Cs_0.2_FA_0.8_Pb(I_0.8_Br_0.2_)_3_ at low temperature, which crystallizes preferentially when the substrate temperature is increased. Potential amorphous phases would also agree with the less sharp absorption onset for substrate temperature ≤0 °C (Fig. 1a). Importantly, the peaks shift to higher angles when the substrate temperature is increased (Fig. 1e), confirming that the blue-shift of the bandgap is due to a higher bromide concentration, which is synonymous with a lower FAI content, resulting from its strongly temperature-dependent sticking coefficient. This hypothesis is further supported by the trend in the I/Pb atomic ratio, as estimated by energy-dispersive X-ray spectroscopy (EDX, Fig. S4†), which shows a decrease in iodine content with increasing substrate temperature. Looking at the tendency of the main diffraction peaks' position with increasing substrate temperature (Fig. 1f), one can notice that the trend is again not monotonic, as the peaks corresponding to the sample deposited at 75 °C are at lower angles compared to the 50 °C case. This speaks for a different growth at such high substrate temperature, which will be discussed in the following.

**Fig. 2:**
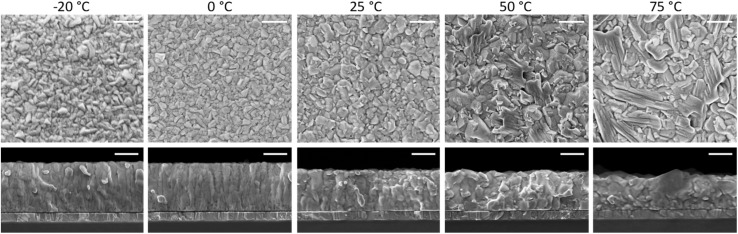
Surface morphology and cross-section SEM images of a series of perovskite films deposited at increasing substrate temperature as noted above. The scale bar corresponds to 300 nm.

At high substrate temperature, the perovskite structure grows highly oriented with respect to the substrate, as only intense and sharp diffraction peaks corresponding to the (110) and (220) planes are observed. Hence the temperature of the substrate during deposition induces profound changes in the crystallization of the perovskite. To investigate if such changes alter also the film morphology scanning electron microscopy (SEM) was carried out on the surface as well as on the cross-section of freshly cleaved perovskite films (Fig. 2). Indeed, the substrate temperature does influence the Cs_0.2_FA_0.8_Pb(I_0.8_Br_0.2_)_3_ morphology at different levels. First, the film thickness is linearly diminishing with increasing substrate temperature (summary in Fig. S5†). This features agrees with the difference in the optical absorption spectra (intensity and sub bandgap interference patterns) shown in Fig. 1a, and with the temperature-dependence of the sticking coefficient discussed above. The sticking coefficient of FAI is expected to increase with lower temperature, as previously observed for MAI.^29,41,47,48^ Hence more FAI is adsorbed at low temperature, leading to thicker films, which might also be responsible for the higher PLQY (Fig. 1b) and low SNR of the corresponding XRD patterns, as noted before (Fig. 1d). The surface SEM shows a clear evolution from a compact morphology at low temperature, composed of small (≤100 nm) grains, to a less homogeneous structure observed at substrate temperature of 50 °C and 75 °C. The perovskite formed at these high temperatures also shows the presence of larger, elongated crystals (especially evident in the sample deposited at 75 °C), not usually observed in co-evaporated perovskite films. As less FAI is present at high substrate temperature, the PbX_2_ precursor might template the growth, forming a scaffold of flat and elongated structures which is typical of CsX/PbX_2_ inorganic films.^32^ The film cross-section also changes radically with increasing substrate temperature. At −20 °C and 0 °C, the films appears very compact and flat, with an apparent columnar growth perpendicular to the substrate. When the substrate temperature is raised to 25 °C, the morphology changes to a more randomly oriented grain structure, with increased surface roughness. This effect is amplified in the films deposited at higher substrate temperature (50 °C and 75 °C), where the grains and aggregates increase in size, leading to rougher perovskite films. It is also worth noting that, in spite of the drastic variation of film thickness (hence most likely FAI content), there is a systematic absence of reflections related to lead or cesium halides in the XRD (Fig. 1d). It is likely that at 75 °C, the FA^+^ content is already sufficient to fully convert PbX_2_ into perovskite, as evidenced by the XRD patterns. Lowering the substrate temperature may enhance FA^+^ adsorption, potentially leading to films with excess FA^+^. This excess is likely related to the more compact, possibly amorphous, morphology observed by SEM and the reduced SNR observed in the corresponding diffraction.

**Fig. 3:**
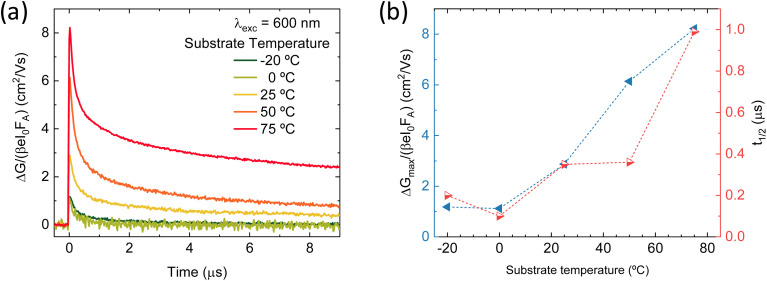
(a) Comparison of TRMC traces of a series of perovskite films vapor-deposited with varying substrate temperature, recorded at 600 nm excitation at the same fluence of 6 × 10^10^ photons per cm^2^ per pulse. (b) Summary of maximum effective mobility (blue, left) and half lifetime (red, right) extracted from the TRMC traces.

These perovskite films were also deposited on quartz substrates at the different substrate temperatures and analyzed by time-resolved microwave conductivity (TRMC, Fig. 3a). Additional TRMC measurements using different laser intensities and different timescales are presented in Fig. S6.† The initial rise of the TRMC signal results from the photogeneration of charge carriers by the short laser pulse and the decay is ascribed to the immobilization of carriers in trap states or by recombination. The maximum TRMC signal corresponds to the product of the sum of the charge carrier mobilities and the generation yield. The lower limit of the mobility is comparable to the values reported in literature for similar vacuum-deposited perovskite films.^56,57^Fig. 3b shows that the TRMC signal magnitude and the lifetime increase with increasing substrate temperature. As TRMC measures the intra-grain charge carrier mobility, this trend mirrors the increasing grain size for higher substrate temperatures shown in the SEM images in Fig. 2. For small perovskite grains, the grains boundaries might limit the observed AC mobility as argued before.^58^ Therefore, the smaller grains can at least partially explain the lower signal size. In addition to that, for the films deposited at low substrate temperatures ≤0 °C, the reduced crystallinity seen by XRD might explain the lower mobility and a corresponding lower TRMC signal.

The same samples series was also analyzed by steady state photocarrier grating (SSPG). For this, coplanar metal electrodes (0.5 mm spacing, 5 mm width, ensuring a homogenous electric field) were evaporated onto the perovskite samples grown at the different substrate temperatures. In this technique, the minority carrier diffusion length (*L*_d_) is obtained from the measurement of the steady-state photocurrent produced by a low applied voltage, while the material is illuminated by two monochromatic laser beams of different intensities that interfere between the two electrical contacts deposited on the films. The photoconductivity measurements give information about majority carriers, while the ambipolar diffusion length *L*_d_ is related to the minority carrier concentration.^59^ As shown in Fig. S7,† the trends of both photoconductivity and *L*_d_ are similar, growing (although non-monotonically) for perovskite films deposited at increasing substrate temperatures. The scattering in the trend might be related with surface defects, as SPPG in this configuration gives information on the lateral charge transport, rather than to the transport across the film cross-section.

**Fig. 4:**
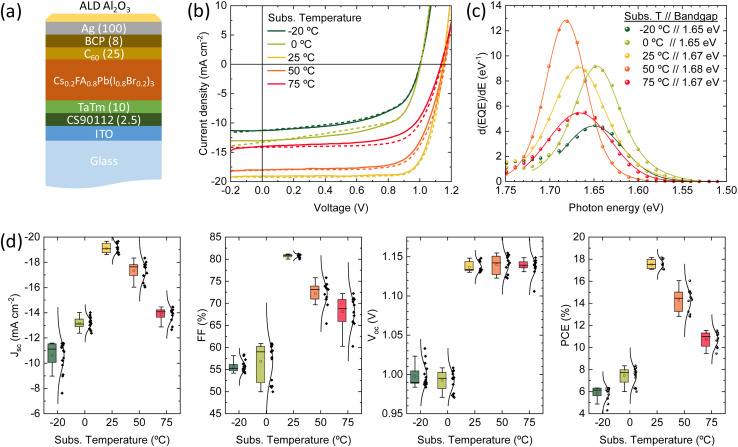
(a) p-i-n device configuration (numbers corresponds to thickness in nm). (b) *J*–*V* curves under simulated solar illumination recorded in forward (from short to open circuit, solid line) and reverse (from open to short circuit, dotted line) bias for representative pixels. (c) Effective bandgap estimation from the derivative of the external quantum efficiency. (d) Statistic distribution of the PV parameters extracted from *J*–*V* curves, for perovskites deposited at increasing substrate temperature.

Finally, we fabricated planar p-i-n solar cells with the structure depicted in Fig. 4a: glass/ITO (120 nm)/CS90112 (2.5 nm)/TaTm (10 nm)/Cs_0.2_FA_0.8_Pb(I_0.8_Br_0.2_)_3_/C_60_ (25 nm)/BCP (8 nm)/Ag (100 nm), where ITO is indium tin oxide, CS90112 is 2,2′,2′′-(cyclopropane-1,2,3-triylidene)tris(2-(*p*-cyanotetrafluorophenyl)acetonitrile), TaTm is *N*,*N*,*N*′,*N*′-tetra([1,1′-biphenyl]-4-yl)[1,1′:4′,1′′-terphenyl]-4,4′′-diamine (TaTm), and BCP is bathocuproine. The Cs_0.2_FA_0.8_Pb(I_0.8_Br_0.2_)_3_ perovskite films were deposited at the same substrate temperatures analysed above. The samples were coated with an alumina film by atomic layer deposition, and the electrical characterization was carried out in ambient atmosphere. Details on the device fabrication and characterization are reported in the Methodology section. The current-density *vs.* voltage (*J*–*V*) curves under simulated solar illumination for a representative solar cell for each deposition temperature are reported in Fig. 4b. The corresponding statistical distribution of the PV parameters as a function of the substrate temperature is reported in Fig. 4d. Fig. 4c presents the first derivative of the EQE spectra, which is used to estimate the effective bandgap energy of the semiconductor within the solar cells. The discrepancies between these values and those derived from the PL spectra in Fig. 1b arise from the fundamental differences between the two techniques: PL provides information solely about the optical properties of the material itself, whereas EQE reflects the full device behavior, including carrier generation, transport, and collection. As such, the EQE-derived bandgap is influenced by both the absorber and the device architecture, often capturing other contributions and effects not visible in PL measurements, such as thickness.^60^ The *J*–*V* curves showed pronounced differences depending on the substrate temperature used for the perovskite deposition. First, for perovskites deposited at temperature other than 25 °C, we do observe a small hysteresis between the forward (from short to open circuit) and reverse (from open to short circuit) voltage scans. This is not typically observed for vacuum deposited solar cells, and indicates the presence of mobile ions in combination with charge carrier recombination sites (see discussion below for details).^61,62^

As the deposition process (precursors deposition rates and their ratio) was optimized for substrate at RT, it is not surprising that the corresponding devices are the more efficient within the series. With a bandgap of 1.67 eV (Fig. 4c), they showed (average values) short circuit current density (*J*_sc_) of 19.1 mA cm^−2^, open-circuit voltage of 1.14 V and a fill factor (FF) of 81%, resulting in a PCE of 17.5%, with record pixels at 18.1%. These parameters are similar or even higher compared to best-in-class wide perovskite solar cells fabricated by vacuum deposition, with similar composition and bandgap.^63^ At lower substrate temperatures, the *J*_sc_ is substantially reduced (10.6 and 13.1 mA cm^−2^ for films grown at substrate temperatures at −20 °C and 0 °C, respectively). For these devices the FF and *V*_oc_ are much reduced compared to RT deposited reference (approximately 55% and 1.00 V). As a result of these parameters, the PCE is low 6–8%, indicating hindered charge extraction and a high rate of non-radiative recombination. For the solar cells containing the films deposited at substrate temperatures above RT (50 °C and 75 °C), we also observe a decrease of the overall power output of the solar cells, although less pronounced as compared to the low-temperature deposited ones. The *J*_sc_ decreases to 17.3 and 13.8 mA cm^−2^, and the FF to 72% and 68%, when the temperature of the substrate is raised to 50 °C and 75 °C, respectively. Interestingly, the average *V*_oc_ for solar cells with perovskite deposited from 25 °C to 75 °C is unvaried, in spite of the trends observed for the PLQY and TRMC discussed above.

Profound information is embedded within *J*–*V* curves, especially when measured at multiple illumination intensities. The dominant recombination loss can be identified when simultaneously fitting *J*–*V* curves at multiple light intensities with drift-diffusion software.^64^ For this purpose, we employ the open-source drift-diffusion software SIMsalabim.^65,66^

A list of all input parameters used in the SIMsalabim simulations can be found in the ESI.† The free parameters are listed in Table 1. Due to the presence of hysteresis in the *J*–*V* curves, bulk traps, interface traps, ion concentration, and ion mobility are set as free parameters, in addition to the electron and hole mobilities.

**Table 1:** Free parameters used in the drift-diffusion software, to simulate simultaneously the *J*–*V* curves at three illumination intensities for the devices containing perovskite films deposited at increasing substrate temperatures

Parameter	Symbol	Value per substrate temperature					
		−20 °C	0 °C	25 °C	50 °C	75 °C	
**Transport in perovskite**							
Electron mobility	*μ*_n_	8 × 10^−5^	4 × 10^−4^	6 × 10^−4^	8 × 10^−4^	2 × 10^−4^	m^2^ V^−1^ s^−1^
Hole mobility	*μ*_p_	2 × 10^−4^	4 × 10^−4^	1 × 10^−4^	1 × 10^−4^	1 × 10^−4^	m^2^ V^−1^ s^−1^

**Trapping of electrons and holes**							
Bulk trap density	*N*_t,bulk_	2.5 × 10^22^	2.3 × 10^22^	3.5 × 10^22^	1.1 × 10^23^	1.4 × 10^23^	m^−3^
HTL/perovskite interface trap density	*N*_t,int,p_	1 × 10^17^	1 × 10^18^	5 × 10^15^	5 × 10^15^	5 × 10^15^	m^−2^
Perovskite/ETL interface trap density	*N*_t,int,n_	2 × 10^17^	5 × 10^17^	5 × 10^15^	1 × 10^15^	3 × 10^15^	m^−2^

**Ions**							
Anion concentration	*N*_anion_	1 × 10^22^	1 × 10^22^	5 × 10^23^	5 × 10^23^	5 × 10^23^	m^−3^
Cation concentration	*N*_cation_	4 × 10^22^	5 × 10^22^	5 × 10^23^	5 × 10^23^	5 × 10^23^	m^−3^
Anion mobility	*μ*_anion_	5 × 10^−11^	5 × 10^−11^	5 × 10^−11^	5 × 10^−11^	3 × 10^−13^	m^2^ V^−1^ s^−1^
Cation mobility	*μ*_cation_	5 × 10^−11^	5 × 10^−11^	5 × 10^−11^	5 × 10^−11^	7 × 10^−13^	m^2^ V^−1^ s^−1^

**Fig. 5:**
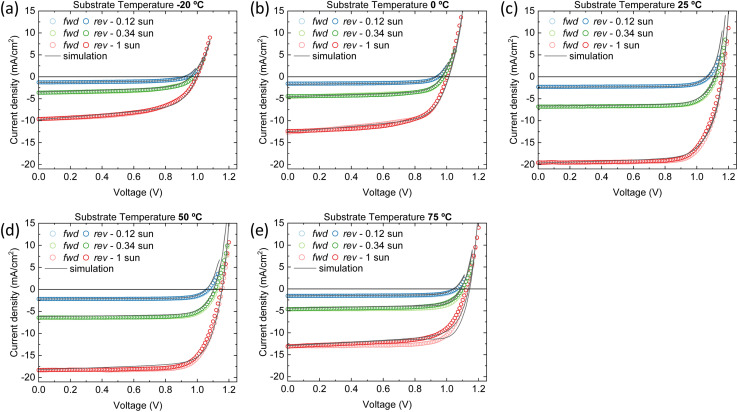
*J*–*V* characteristics of the vacuum deposited wide bandgap perovskite solar cells for multiple light intensities and at (a) −20 °C (b) 0 °C (c) 25 °C (d) 50 °C and (e) 75 °C substrate temperatures. The open symbols represent the experimental data; the solid lines the simulations. First the forward scan from *V*_min_ to *V*_max_ is performed and afterwards the backward scan from *V*_max_ to *V*_min_.

A good agreement between the experimental and simulated *J*–*V* curves is found with these parameter sets, as can be seen in Fig. 5. Therefore, we are able to propose the reason(s) behind the superior performance of the device that contains the perovskite film deposited at 25 °C substrate temperature (hereafter referred to as the ‘best device’). Additionally, we will examine potential improvements for the other devices to ascertain whether the optimal performance at 25 °C is a result of long-term optimization of the deposition process at this temperature or if it is inherently superior.

**Fig. 6:**
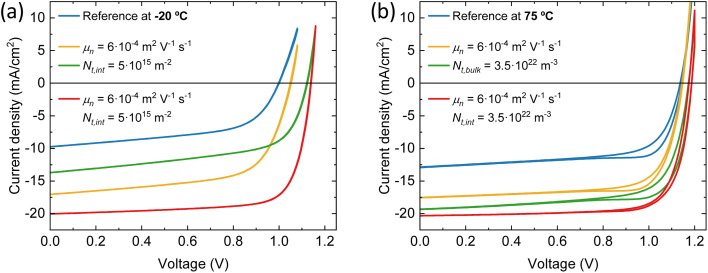
Simulated *J*–*V* curves at 1 sun eq. at (a) −20 °C substrate temperature for different electron mobility and trap density at both interfaces (b) 75 °C substrate temperature for different electron mobility and bulk trap density.

Distinct recombination mechanisms appear to dominate the performance of the devices employing perovskite films deposited below and above room temperature. According to the drift-diffusion simulations, the devices with the perovskite deposited below 25 °C substrate temperature are significantly hindered by low electron mobility and high interface trap densities. For instance, when comparing the device with the perovskite deposited at −20 °C substrate temperature to the best device, a good agreement with the experimental data is obtained only when the electron mobility is reduced by approximately an order of magnitude. If the electron mobility is set to the same value as the best device, the *J*_sc_ increases to 17.0 mA cm^−2^. The significant drop in *V*_oc_ from 1.15 to 1.0 V for these devices, respectively, is explained by an increase in the interface trap density at both transport layers. Fig. 6a shows that removing these interface traps would improve the *V*_oc_ to 1.12 V. When enhancing the electron mobility and removing the interface traps at the same time, the device efficiency would surpass that of the best device. The PCE improves from 16.5% to 17.3%.

The devices with the perovskite films prepared above 25 °C substrate temperature, are hindered by bulk traps, lower ions and electron mobility. The ion mobility of the devices with the perovskite film deposited at 75 °C substrate temperature is two orders of magnitude lower than all other devices in this series. This is not surprising, as only than the experimental *J*–*V* data shows profound hysteresis. Here, the ions are too slow to keep up with the scan speed of 0.1 V s^−1^. As expected, when the ion concentrations are decreased or the ion mobilities are increased, the hysteresis disappears (see Fig. S8†). Additionally, the *J*–*V* curve remains largely unaffected in terms of shape, *J*_sc_, *V*_oc_ and FF. In other words, considering ion concentration and mobility will not enhance device performance, except for reducing hysteresis. On the other hand, when the bulk trap density is decreased, the *J*_sc_ improves significantly, from 12.9 to 19.3 mA cm^−2^ (see Fig. 6b). The same happens for the electron mobility, but to a lesser extent. The *J*_sc_ becomes 17.6 mA cm^−2^. Also, with improved electron mobility, the hysteresis is reduced, whereas reducing the bulk trap density does not have the same effect. When both parameters are improved simultaneously, the resulting *J*_sc_ does not represent a superposition of the individual improvements. This suggests that beyond this point, another factor starts to constrain the *J*_sc_, likely absorption losses. This is indicated by the UV-vis spectra in Fig. 1a that shows that the optical absorbance reduces for films prepared at substrate temperatures from 25 to 75 °C. Yet, this is not incorporated in the simulations, which may result in an underestimation of the electron mobility at 75 °C substrate temperature. The electron mobility of the devices employing the perovskite deposited at 50 °C substrate temperature, surpasses that of the best device. Additionally, the effective mobility continues to increase in the TRMC measurements for devices using perovskite deposited at substrate temperatures of 75 °C. In any case, the drop in *J*_sc_ is attributed to a combination of factors that all point to the fact that the perovskite itself limits the device performance. These factors are absorption losses, higher bulk trap density, lower ion concentration, lower ion and electron mobility. Similarly, for devices using perovskites prepared at substrate temperatures below 25 °C, the substantial increase in the number of interface traps on both sides, coupled with the low electron mobility, indicates that the perovskite active layer is the limiting factor and not either of the charge extraction layers. That the perovskite is the limiting factor and not the HTL and/or ETL, even for interface traps, is because the interface trap density increases on both sides. Therefore, at the HTL/perovskite and perovskite/ETL interfaces, the perovskite compatibility seems poor.

In view of the high charge carrier mobility (obtained by TRMC and confirmed by simulations) and the long recombination lifetime, the perovskite films deposited with substrate temperature of 75 °C are very promising for application in solar cells. In order to understand their inferior device performance, we have deposited the perovskite at 75 °C in the same run on different substrates: glass, glass/ITO and glass/ITO/TaTm. As evident in Fig. S9a,† the optical absorption profiles suggest that the material formation at this substrate temperature is strongly dependent on the underlying layer. While on glass the material shows a steep optical absorption and intense diffraction pattern (Fig. S9b and c†), on ITO and TaTm both the absorbance close to the bandgap and the SNR of the diffraction patterns are much lower. As our solar cells use ITO/TaTm as the substrate for the perovskite deposition, it is not surprising that the devices using the perovskites deposited at high substrate temperature underperform compared to those based on the perovskites deposited at RT. As the TRMC analysis are carried out on perovskite films deposited on silica, hence the higher mobility observed with this technique. The origin of the strong dependence on the substrate surface for the perovskite grown at 75 °C is not fully understood. At high temperature, the FAI adhesion would be hindered, even more on a non-polar surface such as TaTm. We did attempt to improve the perovskite formation at this temperature by reducing the deposition rates of the cesium and lead precursors (nominally increasing the FAI content), but only marginal differences were observed when the perovskite was deposited on TaTm.

**Fig. 7:**
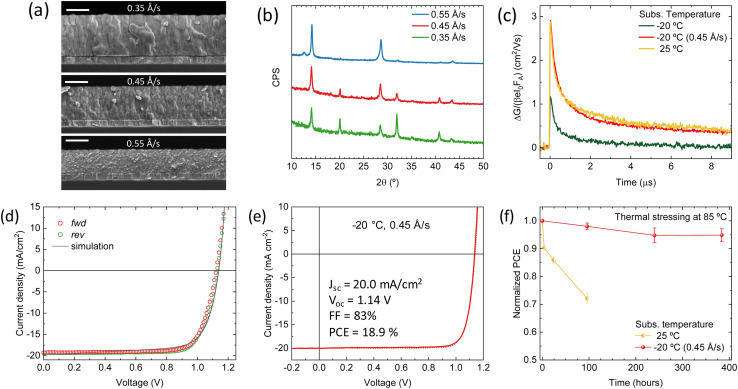
(a) SEM cross-sections (scale bar 300 nm) and (b) XRD patterns of perovskite films obtained with increasing deposition rates of the lead halide precursor, and deposited with substrate temperature of −20 °C. (c) TRMC traces for the perovskite films obtained at different substrate temperatures, with a Pb(I_1−*x*_Br_*x*_)_2_ deposition rate of 0.35 Å s^−1^ and 0.45 Å s^−1^ for the optimized samples at −20 °C, recorded for above-bandgap excitations at the same fluence of 5–8 × 10^10^ photons per cm^2^ per pulse. (d) *J*–*V* curve at −20 °C substrate temperature optimized for the Pb(I_1−*x*_Br_*x*_)_2_ deposition rate (0.45 Å s^−1^). The open symbols represent the experimental data; the solid lines the simulations. (e) *J*–*V* curve under simulated solar illumination for the record device obtained with the substrates and (f) comparison of the thermal stability of devices obtained at 25 °C and −20 °C.

From the point of view of the morphology, the perovskite films deposited at low substrate temperature (−20 °C) are also interesting, as the film cross section appears very compact with a smooth surface (Fig. 2), which is beneficial for optoelectronic devices. For this reason, we modified the deposition rates for this substrate temperature, in an attempt to recover the device functioning. In this case, as suggested by the thickness and the temperature-dependent adhesion of organic materials, a higher incorporation of FAI is expected at low temperature. In order to tune the perovskite composition deposited at −20 °C, we hence increased the Pb(I_1−*x*_Br_*x*_)_2_ deposition rate from 0.35 Å s^−1^ to 0.45 Å s^−1^ and 0.55 Å s^−1^, keeping the rest of the process unchanged. As the process is terminated when the lead halide sensor reading reaches 280 nm, increasing its rate results in a lower degree of FAI intake and hence thinner films, as confirmed by SEM cross-section (Fig. 7a).

The compact structure is maintained at this substrate temperature, although a more granular texture appears when the Pb(I_1−*x*_Br_*x*_)_2_ deposition rate is increased, especially evident at the highest rate. The diffraction patterns (Fig. 7b) also change accordingly, with a reorientation of the main reflections indicating a preferential orientation along the *b*-axis (perpendicular to the substrate), as evidenced by the two main reflections at 2*θ* = 14.2° and 2*θ* = 28.6° observed for the samples deposited at 0.55 Å s^−1^. This sharpening and intensification of the (100) and (200) peaks suggest a strong (100) texture, indicating that higher deposition rates promote more oriented crystalline growth.

The PL intensity was found to diminish and the peak to blue-shift with higher Pb(I_1−*x*_Br_*x*_)_2_ deposition rate (Fig. S10a†), the latter obviously caused by the higher bromide content (also observed from the optical absorption in Fig. S10b†). Solar cells with the same p-i-n structure as described above were prepared for this series of perovskites prepared at substrate *T* = −20 °C and with different Pb(I_1−*x*_Br_*x*_)_2_ deposition rates (Fig. S11†). We obtained improved and high performing devices for Pb(I_1−*x*_Br_*x*_)_2_ deposition rate of 0.45 Å s^−1^. When further increasing the deposition rate of Pb(I_1−*x*_Br_*x*_)_2_ the device performance is reduced. On average, the best devices containing the perovskites obtained at the 0.45 Å s^−1^ deposition rate of Pb(I_1−*x*_Br_*x*_)_2_ showed *J*_sc_ of 19.1 mA cm^−2^, *V*_oc_ = 1.12 and FF = 78%, resulting in a PCE of 16.8%, which is close to that of the devices prepared with perovskites deposited at room temperature. These optimized perovskite films deposited at −20 °C were further analyzed by TRMC as shown in Fig. 7c and S12.† Most interestingly, a substantial increase in signal intensity and lifetime is visible upon optimization of the deposition rate. The obtained signal is comparable to the perovskite film deposited on the substrate at 25 °C. Moreover, from Fig. S12,† it can be concluded that for the optimized sample more second order recombination is present, as deduced by the enhanced intensity-dependent behavior than observed in the original sample. This confirms the beneficial effect of optimizing the FAI rate.

The predicted improvements in device parameters from the drift-diffusion simulations are confirmed, particularly the increase in electron mobility and the reduction in interface trap densities (see Table S3† and Fig. 7d). However, a slight increase in bulk trap density and a reduction in hole mobility is observed, though these factors have a minimal impact on the overall solar cell performance. In fact, both the *J*_sc_ and *V*_oc_ are very close to those of the devices containing perovskites prepared at substrate room temperature (25 °C). This finding is corroborated by the TRMC measurements, where the effective mobility of the optimized perovskite (−20 °C) is nearly identical to that of the perovskite prepared at a substrate at room temperature. The main difference between the device parameters of the optimized device and device prepared with substrate at room temperature, is that the former has a reduced ionic concentration in the perovskite (as the original device at −20 °C substrate temperature had less ions). This result suggests that using lower substrate temperatures during fabrication may reduce the mobile ion concentration in the perovskite, favoring device functioning.

Using the optimized deposition rate of 0.45 Å s^−1^ for Pb(I_1−*x*_Br_*x*_)_2_ at a substrate temperature of −20 °C, we increased the perovskite film thickness to approximately 750 nm and integrated these layers in solar cells. This led to devices with *J*_sc_ up to 20.0 mA cm^−2^ and PCE = 18.9% (record pixel in Fig. 7e). These are among the highest performing fully vacuum-deposited solar cells reported to date for a bandgap of 1.65 eV. We evaluated the stability of these devices and compared them to reference solar cells containing perovskites produced with the substrate at room temperature. Devices were continuously stressed at 85 °C in the dark, periodically measuring their *J*–*V* curves to extract the PCE. As reported in Fig. 7f, the reference devices were not thermally stable, decaying to 70% of the initial PCE after 4 days at 85 °C. On the other hand, solar cells with perovskites deposited at low substrate temperature (−20 °C) showed a much improved thermal stability, with *t*_95_ (time to reach 95% of the initial PCE) of 16 days (384 hours). Stressing the same devices under illumination accelerates the performance decay (*t*_95_ of 6 days, Fig. S13†). However, the solar cells using perovskites deposited with substrate temperature −20 °C retains 80% of the initial PCE after 600 hours under illumination at 65 °C. We ascribe the stability enhancement to the more compact morphology that can be attained with our deposition process at low substrate temperature, leading to less defect (ionic) density, as also confirmed by the numerical simulations.

## Conclusion

We have investigated the influence of the substrate temperature (−20 °C to 75 °C) on the properties of vacuum deposited multicomponent Cs_0.2_FA_0.8_Pb(I_0.8_Br_0.2_)_3_ wide bandgap perovskite. The morphology of the films is found to be profoundly affected by the deposition temperature. These changes entail variations in the material properties, both optical and electronic. By time-resolved microwave conductivity, we observed an increase of the effective mobility of the mobile charge carriers and a longer recombination lifetime when increasing the substrate temperature from −20 °C to 75 °C, in line with increase in grain size at higher temperature. For lower substrate temperatures, the carrier transport properties are thought to be limited by the small grains and excess of FAI at grain boundaries. Although these properties are not directly translated in an improved device functioning, drift-diffusion simulations also confirmed the increased effective mobility, but also highlights other loss mechanisms responsible for the performance of perovskite diodes. At substrate temperatures below 25 °C, low electron mobility in combination with high interface trap densities significantly hinder the device performance. Above 25 °C, bulk traps, electron mobility, ions, and absorption losses are the limiting factors. These simulation results suggest the perovskite layer itself constrains the overall performance. Indeed, when optimizing the deposition rates and using a substrate temperature of −20 °C, we are able to obtain wide bandgap perovskite solar cells with enhanced performance and also improved thermal stability. This work demonstrates the potential of the substrate temperature during as an additional and important parameter to tune the deposition process towards better quality materials.

## Methodology

### Materials

TaTm, CsI and PbBr_2_ were obtained from Tokyo Chemical Industry. CS90112 and PbI_2_ were purchased from Luminescence Technology Corp. FAI was obtained from Greatcell Solar Materials. Fullerene (C_60_) was obtained from Merck KGaA.

### Film and device preparation

ITO-coated glass substrates were subsequently cleaned with soap (2% Mucasol™ in water), water and isopropanol in an ultrasonic bath, followed by 20 min UV-ozone treatment. The substrates were transferred to a vacuum chamber integrated in a nitrogen-filled glovebox and evacuated to a pressure of 10^−6^ mbar for the charge extraction layers' deposition. In general, the deposition rate for the TaTm and C_60_ was 0.5 Å s^−1^ while the thinner CS90112 and BCP were deposited at 0.2 Å s^−1^. Ag was evaporated in a separate vacuum chamber using aluminum boats as sources. The perovskite was evaporated in a dedicated vacuum chamber, equipped with four evaporation sources (M. Braun Inertgas-Systeme GmbH) with independent temperature controllers and shutters. All sources have a dedicated QCM sensor above and the materials are loaded in alumina crucibles. All sources were individually calibrated for their respective materials and no cross-reading between materials is ensured by the relative position of the sources, shutters and sensors. The mixed halide Pb(I_1−*x*_Br_*x*_)_2_ precursor was prepared by mixing in an alumina crucible the calculated amounts of PbI_2_ and PbBr_2_, and by heating them at 380 °C for 5 minutes, when complete melting of the mixture is achieved. During the Cs_0.2_FA_0.8_Pb(I_0.8_Br_0.2_)_3_ perovskite deposition, the deposition rates of FAI, CsI and Pb(I_1−*x*_Br_*x*_)_2_ were kept constant at 0.45 Å s^−1^, 0.1 Å s^−1^ and 0.35 Å s^−1^. During the perovskite deposition, the pressure of the chamber was maintained at 8 ×10^−6^ mbar. The temperature of the substrates was changed from −20 °C to 75 °C, and the temperature was controlled with an oil chiller connected to the copper sample holder. Typical sublimation temperatures for the precursors were 150 °C for FAI, 260 °C for the Pb(I_1−*x*_Br_*x*_)_2_ mixture and 410 °C for CsI. All devices were coated with Al_2_O_3_ (30 nm) by atomic layer deposition (Arradiance's GEMStar XT Thermal ALD) prior to the characterization, which was carried out in ambient atmosphere.

### Materials characterization

Absorption spectra were collected using fiber optics based Avantes Avaspec2048 Spectrometer. The photoluminescence spectra were measured with an Avantes Avaspec2048 spectrometer and films were illuminated with a diode laser of Integrated Optics, emitting at 515 nm. All spectra were collected with an integration time of 1 s. The XRD patterns were collected in Bragg–Brentano geometry on an Empyrean PANalytical powder diffractometer with a copper anode operated at 45 kV and 40 mA. Scanning Electron Microscopy (SEM) was performed with a high-resolution field-emission Hitachi SU8010 microscope operating at an accelerating voltage of 2 kV over platinum-metallized samples. Steady state photocarrier grating (SSPG) measurements were carried out using a He–Ne laser with 15 mW power and 632 nm wavelength. Samples for SSPG consists in perovskite films deposited on glass, coated with two 5 mm wide Au electrodes separated by a 0.5 mm gap. Using neutral density filters, a generation rate of 3 × 10^21^ (cm^−3^ s^−1^), which is close to 1 sun equivalent intensity, was obtained. Time-resolved microwave measurements were carried out under N_2_ by placing the perovskite films in a sealed microwave cavity cell. Charge carrier photoexcitation was performed by a nanosecond pulsed laser light, at 600 nm with varying laser fluences (between 10^9^ to 10^12^ photons per cm^2^ per pulse) adjusted by using neutral density filter. For probing microwaves in the range 8.2–12.4 GHz is used. The normalized reduction in microwave power due to the interaction with free, mobile carriers was recorded over time, Δ*G*(*t*), according to the relation Δ*P*(*t*)/*P* = −*K*Δ*G*(*t*). The sensitivity factor, *K*, applied to all measurements is 78 000. The maximum TRMC signal is interpreted as the product of the sum of the charge carrier mobilities and the charge carrier yield. All the traces have been normalized for the incident laser intensity, *I*_0_, and the fraction of absorbed light, *F*_A_, at 600 nm. Further information about the instrumental set-up and analyses are provided in previous studies.^67,68^

### Device characterization

*JV* curves were recorded using a Keithley 2612A SourceMeter in a −0.2 and 1.2 V voltage range, with 0.01 V steps and integrating the signal for 20 ms after a 10 ms delay, corresponding to a scan speed of about 0.1 V s^−1^. The devices were illuminated under a Wavelabs Sinus 70 LED solar simulator. The light intensity was adjusted before every measurement using a calibrated Si reference diode. The active area, defined as the overlap between the bottom ITO and the top metal electrodes, was 5.5 × 1.5 mm^2^. For the characterization under illumination, a shadow mask defining an area of 5 × 1 mm^2^ was used. For the sensitive EQE measurements, the cell was illuminated by a Quartz-Tungsten-Halogen lamp (Newport Apex 2-QTH) through a monochromator (Newport CS130-USB-3-MC), a chopper at 279 Hz and a focusing lens. The device current was measure as a function of energy from 2.1 eV to 1.2 eV in 0.02 eV steps using a lock-in amplifier. The system was calibrated and the solar spectrum mismatch was corrected using a calibrated silicon reference cell. Fast EQE measurements were performed on a QE-R system from Enlitech.

### Simulations

A list of all input parameters can be found in the ESI.† The experimentally-obtained thickness and bandgap of the perovskite films were used as input parameters. Material optics are also incorporated in the drift-diffusion simulations by using the transfer matrix model.^69,70^ For this purpose, the solar simulator spectrum as specified by the manufacturer is obtained, and the complex refractive index of TaTm and the perovskite at a substrate temperature of −20 °C is determined through ellipsometry (see Fig. S9†). The complex refractive index of C_60_ is obtained from the literature.^71^

## Conflicts of interest

There are no conflicts to declare.

## Supplementary Material

EL-001-D5EL00021A-s001

## Data Availability

Data from the manuscript will be made available at the University of Valencia's repository Roderic (https://roderic.uv.es/) and European repository Zenodo (https://zenodo.org/).
