# Supplementary material for: Tuning substrate temperature for enhanced vacuum-deposited wide-bandgap perovskite solar cells: insights from morphology, charge transport, and drift-diffusion simulations
Source: EES Solar. 2025 May 14;1(3):391–403. doi: 10.1039/d5el00021a (PMC12093137; doi:10.1039/d5el00021a)
Supplement: EL-001-D5EL00021A-s001 [file EL-001-D5EL00021A-s001.pdf]

Supplementary Information (SI) for EES Solar.
This journal is © The Royal Society of Chemistry 2025

SUPPORTING INFORMATION

Tuning Substrate Temperature for Enhanced vacuum-deposited Wide-Bandgap Perovskite Solar Cells: Insights from Morphology, Charge Transport, and Drift-Diffusion Simulations

Lidón Gil-Escrig, Jasmeen Nespoli, Fransien D. Elhorst, Federico Ventosinos, Cristina Roldán-Carmona, L. Jan Anton Koster, Tom Savenije, Michele Sessolo*, Henk J. Bolink*

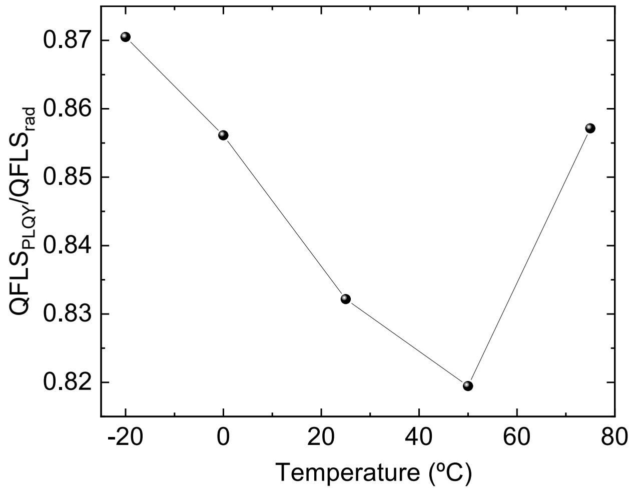

Figure S1. Ratio of the QFLS calculated from the PLQY ($QFLS_{PLQY}$) to the QFLS in the radiative limit ($QFLS_{rad}$) for the series of perovskites deposited at increasing substrate temperature.

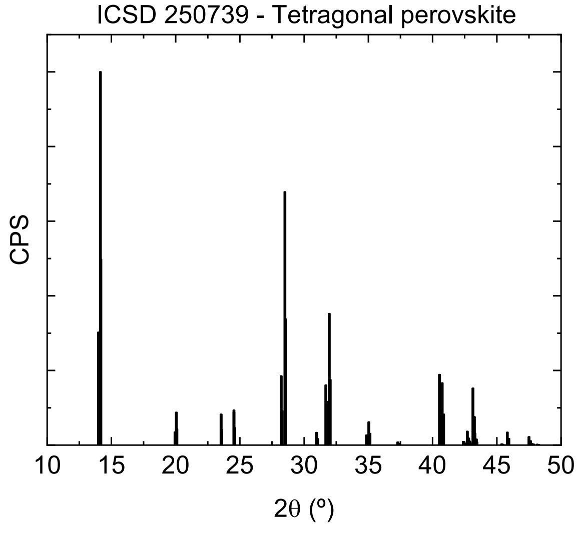

Figure S2. Reference XRD pattern for a tetragonal perovkite obtained from the Inorganic Crystal Structure Database (ICSD collection code 250739).

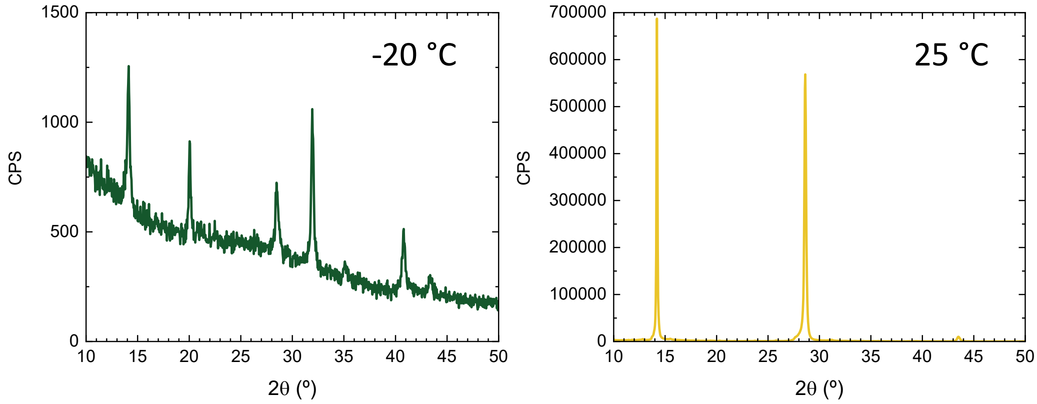

Figure S3. Raw un-normalized XRD data for a perovskite film deposited at −20 °C (left) and for one deposited at RT (right).

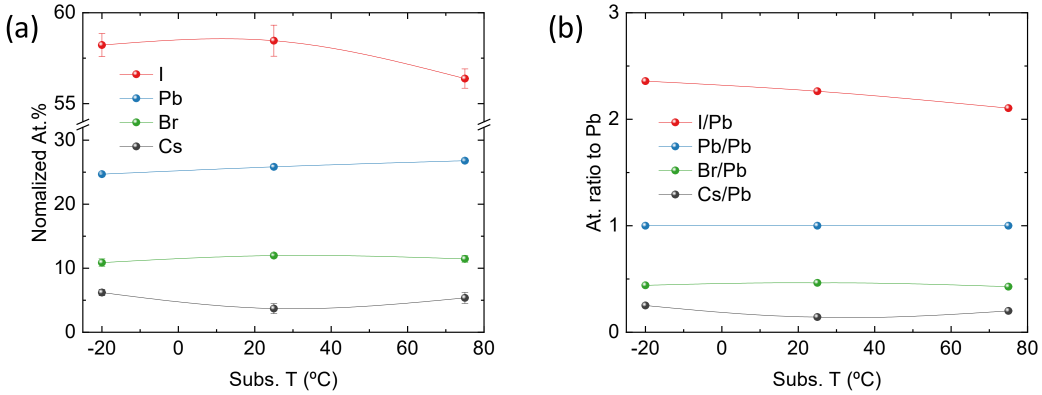

Figure S4. (a) Normalized atomic concentration for selected elements (Cs, Pb, I, Br) estimated from EDX of perovskite films deposited at varying substrate temperature. (b) Ratio of the same values as in (a) to Pb.

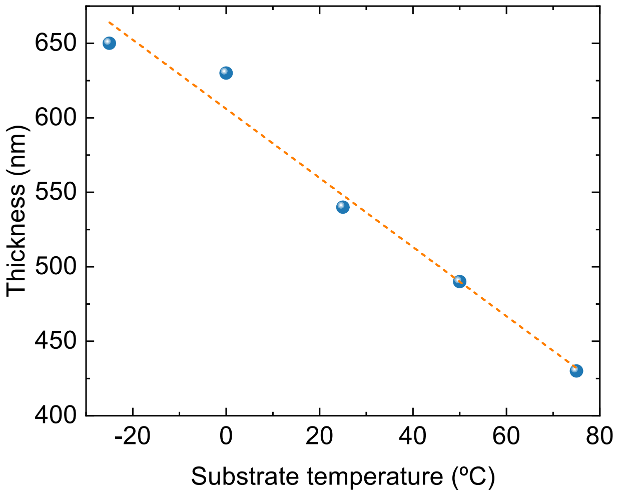

Figure S5. Dependence of the film thickness with the substrate temperature used during vacuum deposition. Thickness is obtained from cross-sectional SEM.

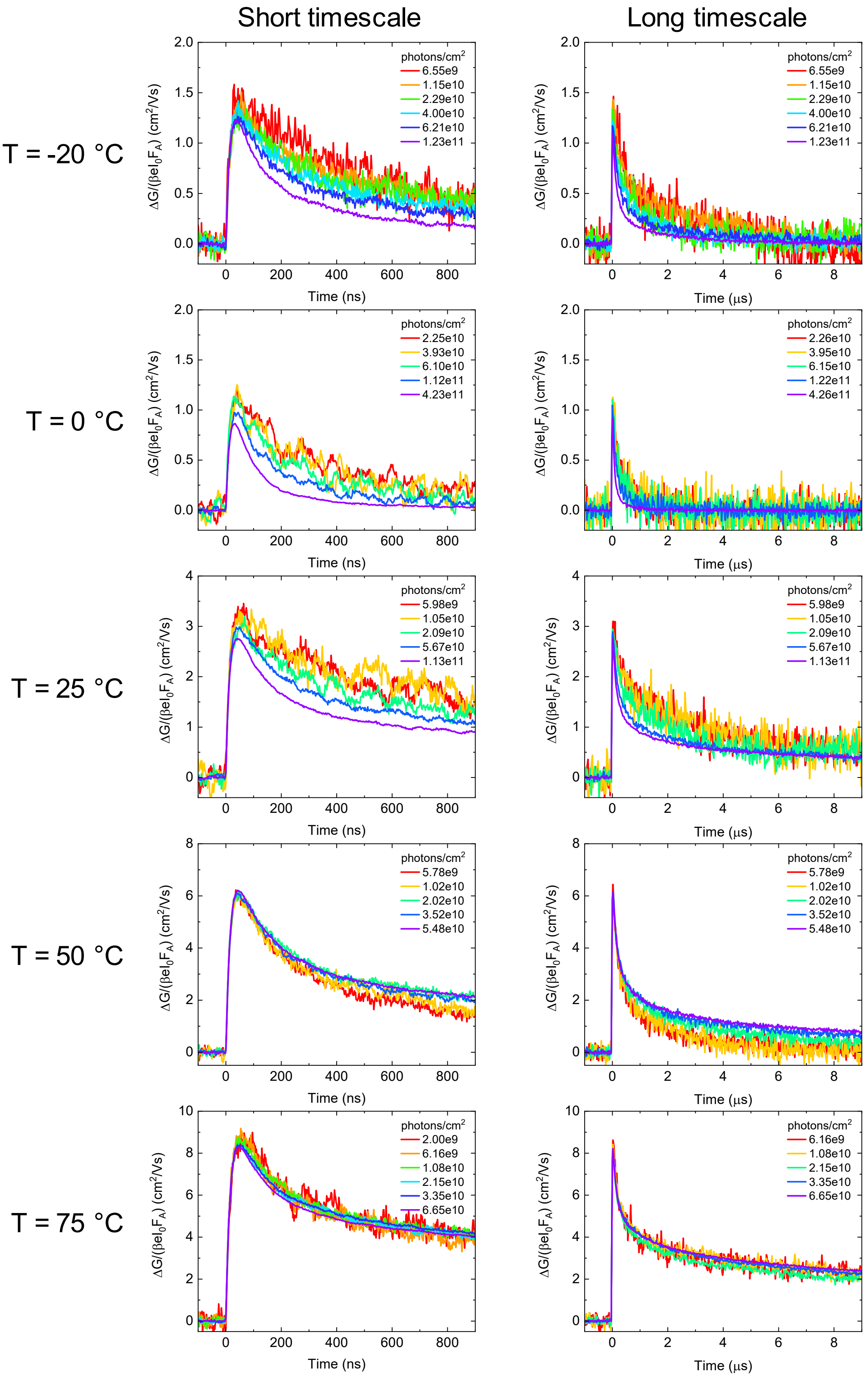

Figure S6. TRMC traces at 1 microsecond timescale (left column) and 10 microsecond timescale (right column) of a series of perovskite films deposited at increasing substrate temperatures as noted, recorded on excitation at 600 nm with varying laser intensities.

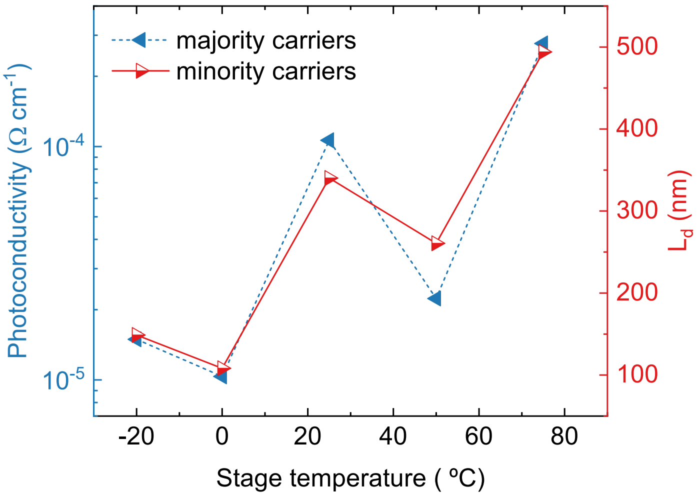

Figure S7. Photoconductivity (left, blue symbols) and minority carrier diffusion length (right, red symbols) measure on perovskite films deposited at increasing substrate temperature.

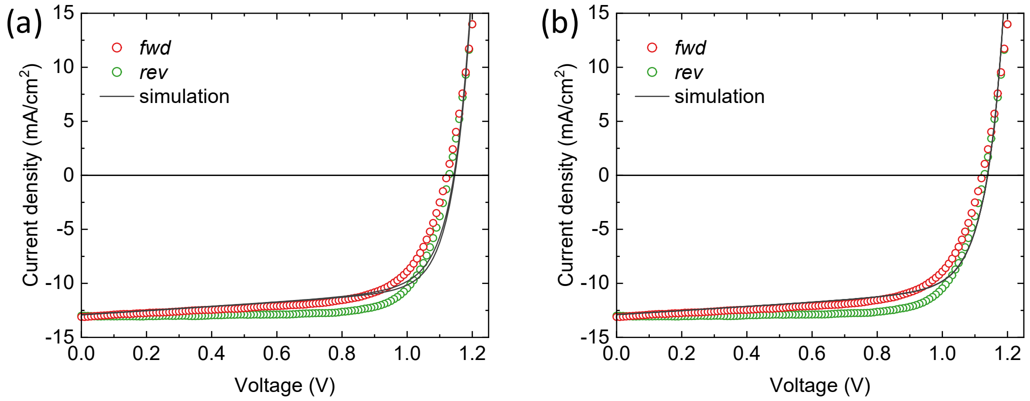

Figure S8. Simulated J-V curve for 75 °C substrate temperature a) when setting the anion and cation concentration to $1\cdot10^{22}$ and $4\cdot10^{22}$ m^{-3}, respectively. These ion concentrations are used in the -20 °C fit. b) When setting both the anion and cation mobility to $5\cdot10^{-11}$ $m^2V^{-1}s^{-1}$. These ion mobilities are used for all the other fits.

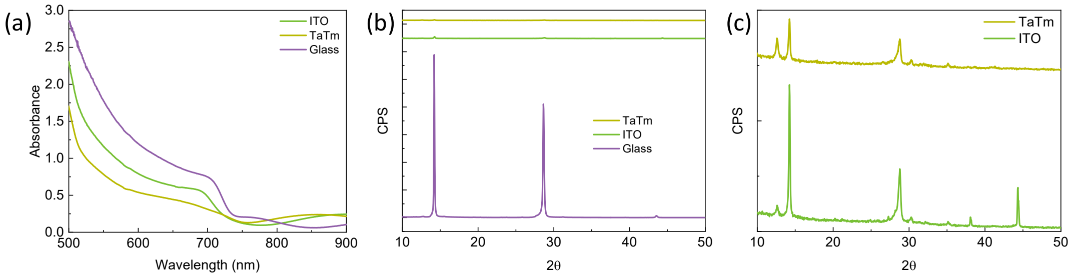

Figure S9. (a) Optical absorption of perovskite films deposited with a substrate temperature of 75 °C on different layers and (b) corresponding XRD patterns. (c) Zoom of the XRD patterns collected on TaTm and ITO.

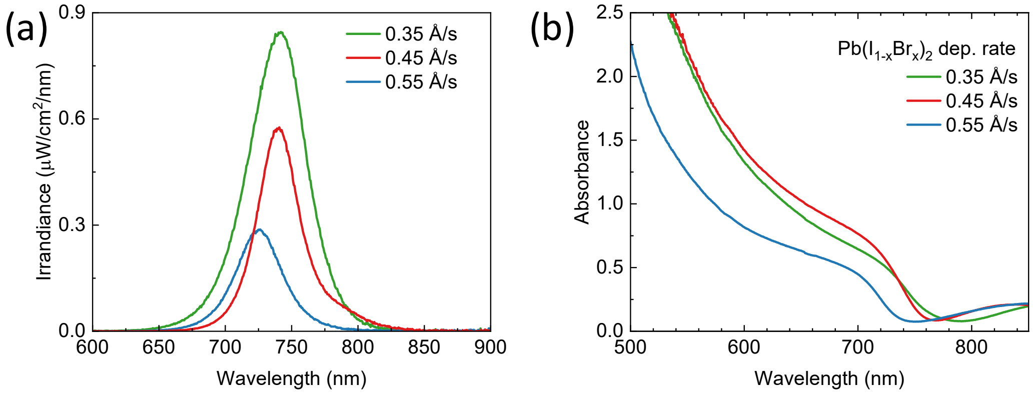

Figure S10. (a) PL spectra and (b) optical absorption of perovskite films deposited with a substrate temperature of −20 °C and with increasing deposition rate of the lead halide precursor.

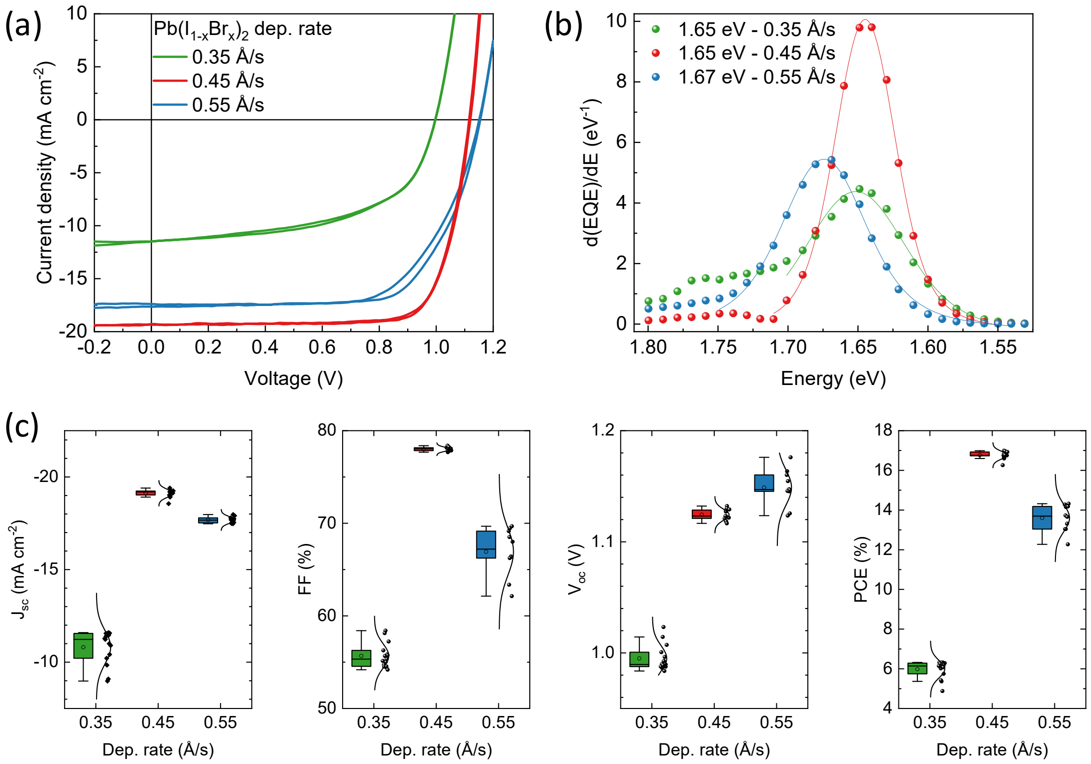

Figure S11. Characterization of solar cells with perovskite deposited with a substrate temperature of −20 °C and with increasing deposition rate of the lead halide precursor. (a) JV curves under simulate solar illumination, (b) first derivative of the EQE spectra used to determine the bandgap) and (c) statistics of the PV parameters.

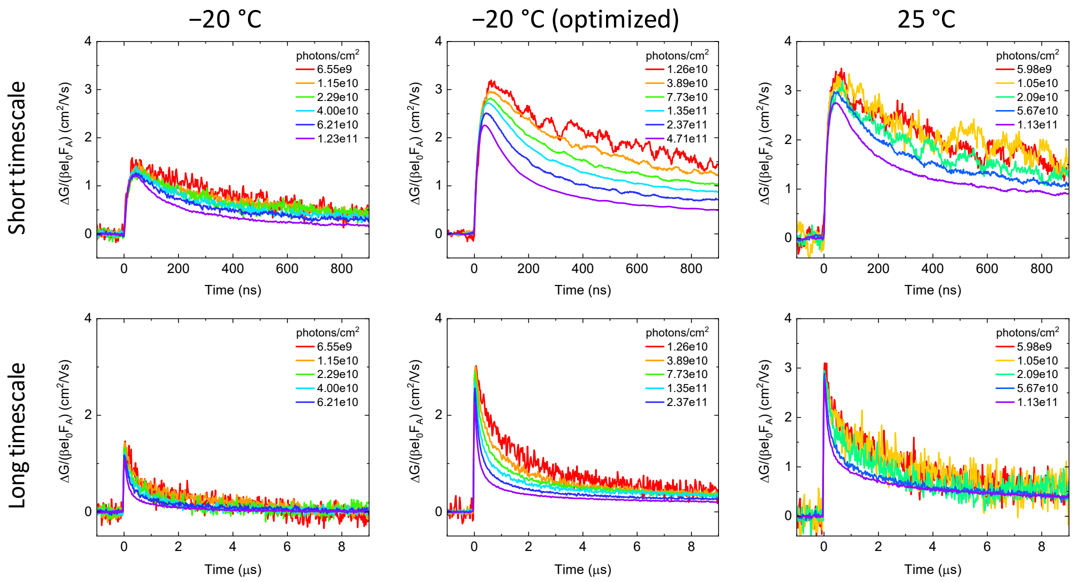

Figure S12. TRMC traces at shorter timescale (first row) and longer timescale (second row) of a series of perovskite films deposited at different substrate temperature and deposition parameters as noted above (columns), recorded for above-bandgap excitations with varying laser intensity. The TRMC traces for substrate temperature at -20ºC (left column) and 25ºC (right column) are the same shown in Figure S3b, with an altered y-axis range.

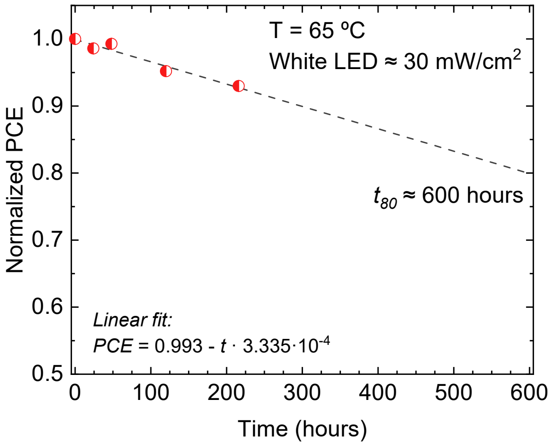

Figure S13. Evolution of the PCE for solar cells using perovskite deposited with substrate at −20 °C (red symbols). Samples are kept at 65 °C and continuously illuminated at open circuit with a white LED array (≈ 30 mW/cm^2). The dash line is a linear extrapolation of the decay.

Input parameters to drift-diffusion simulations

The parameters that remain constant across all simulations are listed in Table S1. The relative permittivities, bimolecular recombination rate, capture coefficients, and mobility in the TLs are retrieved, where possible, from previous drift-diffusion simulations on perovskite solar cells. The thicknesses of TaTm and C_{60} are determined experimentally. The perovskite thickness and bandgap vary with substrate temperature (see Table S2 and Figure S4).

The density of states is computed from the electron effective mass, reported to be 0.1 for $FAPbI_3$ in [1] and 0.17 for $CsPbI_3$ in [2]. As we work with the double cation perovskite $Cs_{0.2}FA_{0.8}Pb(I_{0.8}Br_{0.2})_3$, the average of both values (0.14) is used to obtain an effective density of states of $1.282 \cdot 10^{24}$ m^{-3} (see formula 1).

$$N_{cv} = 2 \cdot \left(\frac{2\pi m_e k_B T_3}{h^2}\right)^{3/2} \quad (1)$$

For ease of comparison between substrate temperatures, the anode work function and HTL HOMO are assumed to perfectly align with the valence band of the perovskite (which varies with each temperature due to the measured differences in bandgap). Similarly, the cathode work function and ETL LUMO are assumed to perfectly align with the conduction band of the perovskite. For all substrate temperatures the perovskite conduction band is fixed to 4 eV, as the conduction band is reported to be around 4 eV for $FA_{1-y}Cs_yPb(I_{1-x}Br_x)_3$ perovskites.[1] Therefore, the valence band changes with the bandgap.
To ensure the HTL effectively blocks electrons, the energy offset between the HTL LUMO and perovskite conduction band is set to 1 eV. Likewise, the ETL HOMO and perovskite valence band offset is set to 1 eV. Lastly, the trap levels are set to the middle of the bandgap.

The illumination intensities of 0.1 and 0.32, as reported experimentally, are slightly increased in the simulations to 0.12 and 0.34 sun eq. This leads to improved correspondence between the simulated and experimental J-Vs, and is considered to be within the experimental error margin.

Table S1. Constant input parameters over all drift-diffusion simulations

parameter	symbol	value	unit	reference
TaTm thickness	d_{TaTm}	12.5	nm	
C_{60} thickness	d_{C60}	27	nm	
TaTm relative permittivity	ε_p	3		[3]
C_{60} relative permittivity	ε_n	3.9		[3]
perovskite relative permittivity	E	20		
density of states in perovskite	N_{cv}	$1.282 \cdot 10^{24}$	m^{-3}	[1], [2]
bimolecular recombination constant	k	$1 \cdot 10^{-17}$	m^3s^{-1}	[4]
electron and hole capture coefficients	C_n, C_p	$1 \cdot 10^{-15}$	m^3s^{-1}	
mobility in transport layers	μ_{TLs}	$1 \cdot 10^{-6}$	$m^2V^{-1}s^{-1}$	ETL: [4]

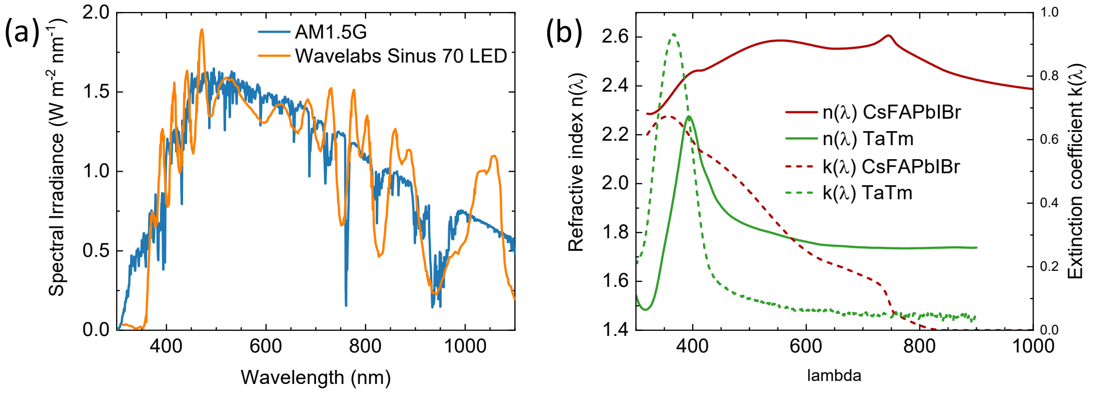

Figure S14. a) The irradiance spectrum of the solar simulator compared to the AM1.5G spectrum. b) The complex refractive index of TaTm and the perovskite at -20 °C substrate temperature as determined with ellipsometry.

Finally, a criterion has to be defined to accept the simulated JV curve and its associated free parameters as representative of the experimental data. For this purpose, the fit-error is applied. The fit-error is the area spanned by the simulated and experimental JV curves divided by their normalization area. The simulated JV curve is accepted when the average fit-error is below 6% (see Table S2).

Table S2. Computed fit-error in percentage between the experimental and simulated J-V curves.

Illumination intensity	Substrate temperature [°C]					
	**-20 optimized**	**-20**	**0**	**25**	**50**	**75**
0.12 sun	NA	4.2	4.2	0.6	1.0	5.3
0.34 sun	NA	8.0	2.5	6.8	9.2	7.4
1 sun	2.4	3.0	2.0	3.4	5.2	5.1
ε_average	2.4	5.1	2.9	3.6	5.1	5.9

Table S3. Parameters used to simulate the optimized device at -20 °C substrate temperature.

symbol	value	unit
μ_n	$2\cdot10^{-4}$	m^2/Vs
μ_p	$2\cdot10^{-4}$	m^2/Vs
$N_{t,bulk}$	$1\cdot10^{22}$	m^{-3}
$N_{t,int,p}$	$1\cdot10^{16}$	m^{-2}
$N_{t,int,n}$	$1\cdot10^{16}$	m^{-2}
N_{anion}	$1\cdot10^{22}$	m^{-3}
N_{cation}	$4\cdot10^{22}$	m^{-3}
μ_{anion}	$5\cdot10^{-11}$	m^2/Vs
μ_{cation}	$5\cdot10^{-11}$	m^2/Vs

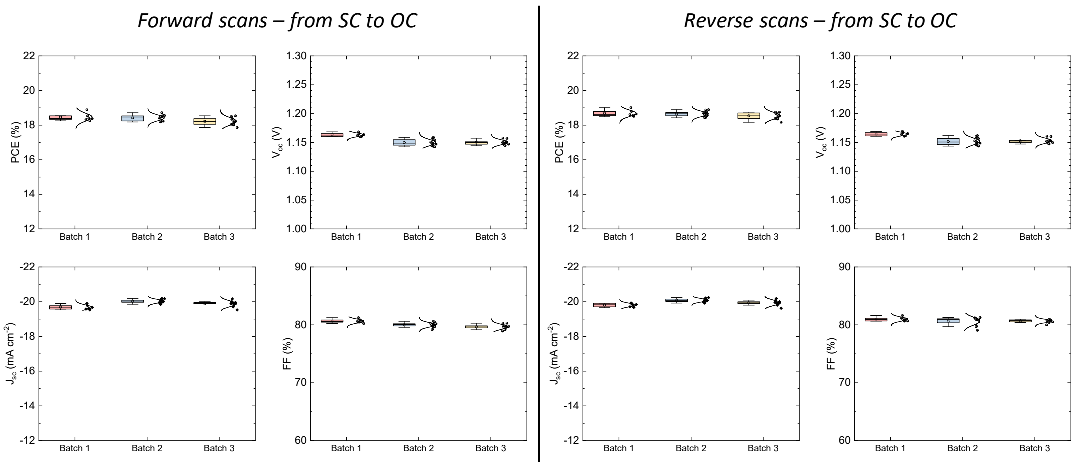

Figure S15. Photovoltaic parameters extracted from J-V curves recorded in forward (left, form short to open circuit) and in reverse (from open to short circuit) bias direction, for three consecutive batches of solar cells using 700-750 nm thick perovskites deposited with substrate temperature at −20 °C.

References

[1] Galkowski, K., Mitioglu, A., Miyata, A., Plochocka, P., Portugall, O., Eperon, G. E., ... & Nicholas, R. J. (2016). Determination of the exciton binding energy and effective masses for methylammonium and formamidinium lead tri-halide perovskite semiconductors. *Energy & Environmental Science*, *9*(3), 962-970. https://doi.org/10.1039/C5EE03435C

[2] Poncé, S., Schlipf, M., & Giustino, F. (2019). Origin of low carrier mobilities in halide perovskites. *ACS Energy Letters*, *4*(2), 456-463.

https://doi.org/10.1021/acsenergylett.8b02346

[3] Sherkar, T. S., Momblona, C., Gil-Escrig, L., Avila, J., Sessolo, M., Bolink, H. J., & Koster, L. J. A. (2017). Recombination in perovskite solar cells: significance of grain boundaries, interface traps, and defect ions. *ACS energy letters*, *2*(5), 1214-1222.

https://doi.org/10.1021/acsenergylett.7b00236

[4] These, A., Koster, L. J. A., Brabec, C. J., & Le Corre, V. M. (2024). Beginner's Guide to Visual Analysis of Perovskite and Organic Solar Cell Current Density–Voltage Characteristics. *Advanced Energy Materials*, 2400055.

https://doi.org/10.1002/aenm.202400055

[5] Prathapani, S., Bhargava, P., & Mallick, S. (2018). Electronic band structure and carrier concentration of formamidinium–cesium mixed cation lead mixed halide hybrid perovskites. *Applied Physics Letters, 112*(9).

https://doi.org/10.1063/1.5016829
